# Running Performance of Male Versus Female Players in Australian Football Matches: A Systematic Review

**DOI:** 10.1186/s40798-021-00391-x

**Published:** 2021-12-19

**Authors:** Christopher Wing, Nicolas H. Hart, Callum McCaskie, Petar Djanis, Fadi Ma’ayah, Kazunori Nosaka

**Affiliations:** 1 School of Medical and Health Sciences, Edith Cowan University, 270 Joondalup Drive, Joondalup, , Perth, WA 6027 Australia; 2 Institute for Health Research, University of Notre Dame Australia, Fremantle, WA Australia; 3 Caring Futures Institute, College of Nursing and Health Sciences, Flinders University, Adelaide, SA Australia; 4 South Fremantle Football Club, Parry Street, Fremantle, WA Australia; 5 School of Education, Curtin University, Bentley, WA Australia; 6 Murdoch Applied Sport Science Laboratory, Murdoch University, Perth, WA Australia

**Keywords:** High-speed running, Match analysis, Microsensor technology, Player Load

## Abstract

**Background:**

Australian Football is a fast paced, intermittent sport, played by both male and female populations. The aim of this systematic review was to compare male and female Australian Football players, competing at elite and sub-elite levels, for running performance during Australian Football matches based on the Preferred Reporting Items for Systematic Reviews and Meta-Analyses (PRISMA).

**Methods:**

Medline, SPORTDiscus, and Web of Science searches, using search terms inclusive of Australian Football, movement demands and microsensor technology, returned 2535 potential manuscripts, of which 33 were included in the final analyses.

**Results:**

Results indicated that male athletes performed approximately twice the total running distances of their female counterparts, which was likely due to the differences in quarter length (male elite = 20 min, female elite = 15 min (plus time-on). When expressed relative to playing time, the differences between males and females somewhat diminished. However, high-speed running distances covered at velocities > 14.4 km·h^−1^ (> 4 m·s^−1^) were substantially greater (≥ 50%) for male than female players. Male and female players recorded similar running intensities during peak periods of play of shorter duration (e.g., around 1 min), but when the analysis window was lengthened, females showed a greater decrement in running performance.

**Conclusion:**

These results suggest that male players should be exposed to greater training volumes, whereas training intensities should be reasonably comparable across male and female athletes.

## Key Points

1. Males complete greater total running distance than female players in Australian Football.
2. When expressed relative to playing time, running distances are reasonably similar between male and female players.
3. Males complete greater total, and relative, high-speed running (> 14.4 km·h^−1^ or > 4 m·s^−1^) than female players

## Introduction

Australian Football (AF) is a fast paced intermittent type sport played on an oval field between two teams of 18 plus 4 players upon the interchange bench amongst elite male players, and between two teams of 16 players with 5 upon the interchange bench within elite female populations [1, 2]. The aim of the game is to successfully transfer the ball through kicks and handballs to create a scoring opportunity, where 6 points are awarded for a goal and 1 point is awarded for a behind (where the ball passes between the inside and outside posts, or hits the inside posts, or where the ball passes between the inside posts having been touched or carried over by another player to the one who had the initial shot) being scored. At the male elite level, the game is played across 4 quarters of 20 min duration plus time on (a period of play added to compensate for all stoppages in play). This time frame differs from the elite female level, where quarters are contested across 15 min, with time on for stoppages included within the final two minutes of each quarter [2]. These playing conditions may differ between elite and sub-elite athletes [3], and may lead to differences in running performance between male and female players. However, no systematic comparison between male and female players has been made.

Players are required to organise into three positional groups at the start of play (i.e., bouncedown) [1]. These are made up of three primary positions, including forwards and backs (half and full positions), as well as a midfield group comprised of inside midfielders, wings (or outside midfielders) and the ruckman (ruck). It is common within research literature to delineate these playing positions into smaller groups [4], or to group them together (e.g., key position players and nomadic players) [5]. This makes cross-study comparisons somewhat challenging [1].

In order for practitioners to develop appropriate training program design and load monitoring protocols, a thorough assessment of player motion during match-play must be undertaken. Wearable microsensor technology is now commonly employed to facilitate this assessment [1]. A microsensor technology device typically consists of a global navigation satellite system (GNSS) as well as a micro-electrical mechanical system (MEMs), which include tri-axial accelerometers, magnetometers and gyroscopes [6]. The GPS component is able to receive signals from orbiting satellites and can provide information upon athlete locomotion and velocity (e.g., total distance travelled) [7–10]. The MEMs component is often utilised to detect match events such as collisions, as well as other measures of motion including accelerations and decelerations [11, 12].

The reliability and validity of these devices have been widely reported within the literature [7–10] and are well summarised in the review by Scott et al. [13]. Specifically, previous research has confirmed both the validity and reliability of GPS technology when using a sampling frequency of 10 Hz, which has been shown to be superior to both 5 Hz [9] and 15 Hz [8] sampling frequencies. However, Johnston et al. [8] raise caution when measuring high velocity movements, as they report that as running speed increases, so does the level of error. Despite this, it should be noted that wearable microsensor technology has enhanced practitioners’ ability to measure athlete motion in team sports, such as AF, and future advances in technology, including local positioning systems (LPS), have the potential to further improve the accuracy, speed and utility of these data collected [14].

There is currently a large body of research concerning the measurement of AF running performance using wearable microsensor technology, reported across a range of metrics (e.g., total distance, high-speed distances), time frames (e.g., full game, quarters), and across various playing levels (e.g., elite, sub-elite), and competitions (male, female). Although it should be noted that systematic reviews focusing on comparisons between male competitors across various playing levels have been published in AF, initially by Gray and Jenkins [15] and more recently by Johnston et al. [1], to the best knowledge of the authors no formal comparisons have been made between male and female AF players, in either reviews or through original research manuscripts, concerning running performance during AF matches. Comparisons of this nature are of increasing importance following the inception of the premier women’s competition (AFLW) in 2017.

This has seen an increased emphasis placed upon developing the sport amongst female players, particularly at the elite level. As the differences in physical and physiological characteristics between male and female athletes are well documented [16, 17], it may be interesting to understand if these are reflected within running performance during AF matches. Additionally, understanding the differences that may exist between male and female players can influence physical training design (e.g., running volumes and intensities), and highlight if there are different requirements between the sexes to transition between the sub-elite and elite levels within their respective developmental pathways, particularly for sport science or strength and conditioning practitioners working across both sexes. Furthermore, if there is a desire to develop the female game into a more high-speed, open game (which is likely considering the recent rule changes (i.e., stand on the mark) to the male game aimed at increasing the “speed of the game”), then comparisons of this type may go some way to highlighting the physical requirements necessary to achieve this. Together, these factors can go some way to influencing the future development of the female game, and in particular, physical performance pathways.

In order to provide a thorough and balanced comparison across the breadth of literature, a systematic review has been conducted with the aim to evaluate the differences in running performance between male and female Australian football players.

## Methods

### Search Strategy

A systematic search of Medline, SPORTDiscus and Web of Science databases, using key terms inclusive of Australian Football, movement demands and microsensor technology, was performed by the lead author (CW) to identify potential peer-reviewed journal articles published in English from inception (Medline and SPORTDiscus, 1988; Web of Science, 1980) until December 2020. Additional publications were also identified through the screening of relevant reference lists. The search strategy was devised through a combination of key words, synonyms and subject headings, as well as through pilot searching of known publications to identify additional relevant terms. The Boolean operators ‘OR’ and ‘AND’ were utilised to construct the final search terms (Table 1).

**Table 1:** Search terms used to identify potential manuscripts

Concept	Search Terms
1. Movement Demands	Movement demands **OR** movement patterns **OR** physical demands **OR** locomotion **OR** running performance **OR** movement profile **OR** match demands **OR** match performance **OR** match play **OR** match characteristics **OR** movement characteristics **OR** activity profiles **OR** game performance **OR** game demands **O**R match play movement **OR** movement **OR** physical exertion **OR** athletic performance **OR** human locomotion **OR** human mechanics **OR** match analysis **OR** movement analysis **OR** acceleration **OR** running **OR** task performance and analysis **OR** athletic ability
2. Australian Football	Australian football **OR** Australian rules football **OR** AFL **OR** football **OR** Australian football players **OR** Australian football league **OR** football players
3. Microsensor Technology	Microsensor technology **OR** global positioning systems **OR** GPS **OR** time motion analysis **OR** global positioning tracking **OR** GPS output **OR** geographic information systems **OR** microtechnology **OR** micro-electrical mechanical systems **OR** accelerometry **OR** global positioning system output

**OR** = the Boolean operator OR

### Screening and Study Selection

Search results were exported to EndNote (X9, Thomson Reuters, Philadelphia, PA, USA), where all duplicates were removed by the lead author (CW). Abstracts and titles were screened by two reviewers (CW, CM), where those that were identified as ‘out of scope’ (including those clearly identified as reviews and commentaries) were removed. Remaining articles were imported into Rayyan [18], an electronic systematic review management tool, where the full texts were independently screened by two reviewers (CW, CM) against the inclusion and exclusion criteria (Table 2). Where disagreement was present, a third reviewer (PD) acted as arbiter. Search findings and study selection are reported in accordance with PRISMA (Preferred Reporting Items for Systematic Review and Meta-analysis) [19].

**Table 2:** Study inclusion and exclusion criteria

Inclusion Criteria	Exclusion Criteria
Original research articles	Reviews, author commentaries, editorials, conference posters/presentations
Competitive able-bodied athletes	GPS system sampling rate < 5 Hz, or GPS or accelerometer sampling rate not reported
GPS system sampling rate ≥ 5 Hz. (Where data were only derived from the accelerometer component sampling rate ≥ 100 Hz)	Non-competitive matches (ie; pre-season) and studies investigating junior players only
Full text available in English	Not in full English
Data only used in one study*	Studies reporting the exact same data sets as previous studies with no additional “new” data
Games played under standard rules for participation level	Missing data sets where average data were used instead
Reports at least 1 measurement of athlete motion for at least 1 specified time period	Studies which examined pre match interventions outside of their normal practice (ie; supplementation, carbohydrate loading) and the impact upon performance
	Distances/ metrics reported only in time and/or percentage spent within a velocity band
	GPS data not reported or are only reported in graphical format
	Age of participants not reported
	Micro-sensor technology metrics not sufficiently defined
	Study only reports combined average data from more than 1 playing level (i.e., combined average for elite and sub-elite)
	Unable to determine playing standard
	Non-GPS or LPS measuring systems (ie; camera tracking)
	Game load not separated from training load
	Studies that investigated tackle counts only
	Studies involving matches played with modified rules/ pitch sizes from those outlined within the rules of the specific competition

*Data from earliest publication used in cases where the exact same data sets were reported

Key: LPS - Local positioning systems; GPS—Global positioning systems

### Data Extraction

Data from articles included within the final review were extracted into a customised Microsoft Excel spreadsheet (Microsoft, Redmond, WA, USA) by the lead author (CW). Data pertaining to sample size (number of matches, subjects, and data files), competition details (type, age-group and playing level), subject demographics (age, height, weight, and sex), measurement duration (e.g., full game, halves, quarters) and measurement approach (e.g., total distance, high-speed running distances, PlayerLoad™, relevant or absolute measures) were recorded. Information regarding the microsensor device (manufacturer, model, software, and sampling frequency (Hz)) and recording accuracy (number of satellites, horizontal dilution of precision (HDOP)) was also recorded in line with recent recommendations [6]. The number of satellite connections is an indicator of GPS signal strength, while the HDOP provides information regarding the accuracy of the horizontal GPS position, with both measures combining to give an indicator of data collection accuracy [6]. Previous research has reported that ≥ 6 satellite connections and a HDOP < 1 are required for optimal data collection accuracy [6].

### Data Analysis

Means for each measure of physical output were recorded and presented within the results section to provide a range. Where comparisons could be made across playing levels, figures were constructed in R software (R, v4.0.3, The R Foundation for Statistical Computing, Vienna, Austria), where the reported means were plotted. High-speed running was also presented as a percentage of total running distances, which were calculated by dividing the mean high-speed distance by the mean total distance.

## RESULTS

### Search Results

**Fig. 1:**
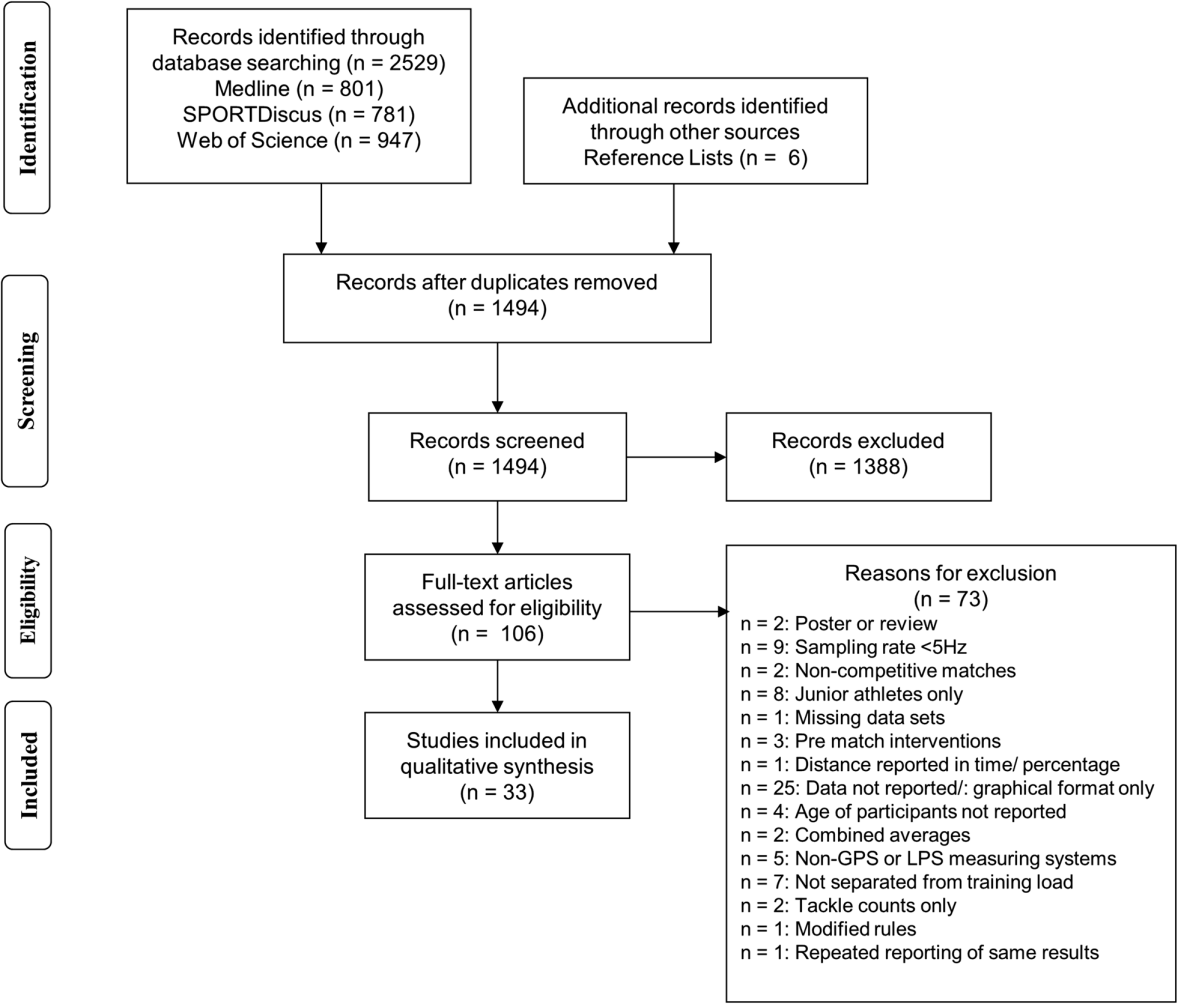
PRISMA flow chart of studies selected for review

The initial search yielded 2529 articles (Medline = 801, SPORTDiscus = 781, Web of Science = 947), with an additional 6 highlighted through searching of the reference lists. From a total of 2535, 1041 were removed as duplicates. Following the screening of the titles and the abstracts, a further 1,388 were removed as out of scope (e.g., the wrong sport), which also included any articles that were author commentaries or reviews. Full texts of the remaining 106 articles were independently screened, with 73 removed according to the exclusion criteria (see Fig. 1). The remaining 33 were included in the final review and analysis.

### Study Characteristics

Characteristics of the included 33 studies are outlined in Table 3. From the included studies, 26 described outcomes for male elite-level, six from male sub-elite level, and one from male amateur or recreational level. Additionally, three studies included female elite level athletes, with a further five studies reporting on female sub-elite level athletes. Although several different microsensor technology metrics were discovered in the literature, only those that could be compared between male and female athletes are discussed within this review. Therefore, this review includes absolute and relative measures of total running distance, high-speed running distances and PlayerLoad™, which were expressed across the whole game, individual quarters, and peak periods of play. Methodological information of the included studies is highlighted in Table 4, of which 26 reported a sampling rate ≥ 10 Hz (with one reporting 5 Hz interpolated to 15 Hz), 9 reported the number of satellite connections, and 10 highlighted the horizontal dilution of precision (HDOP).

**Table 3:** Characteristics of included manuscripts

References	Positions	Sample					Competition Details			
		*n*	Age (years)	Mass (kg)	Height (cm)	Sex	Classification	Year	Matches (*n*)	Files (*n*)
Aughey. [24]	NR	18	25.9 ± 3.5	90.6 ± 8.8	188.4 ± 7.8	M	AFL (elite)	2008/2009	29 1–17 per player	147
Aughey. [61]	Nomadics	8	24.8 ± 3.0	84.5 ± 3.5	184.4 ± 2.7	M	AFL (elite)	2008	6	48
Bellinger et al. [32]	NR	34	22.1 ± 3.3	87.0 ± 8.1	188.2 ± 7.3	M	ALF (elite)	2 seasons	NR	460
Black et al. [41]	MID, HL, FL	22 27	23.2 ± 4.5 23.4 ± 4.9	67.8 ± 8.1 65.4 ± 9	167.2 ± 5 167.9 ± 5	F	QWAFL (sub-elite)	2016	14 4–6 per player	232
Black et al. [39]	MID, HL	43	24.3 ± 5.5	66.5 ± 9.3	167.4 ± 4.3	F	QWAFL (sub-elite)	2016	4–6 per player	180
Black et al. [40]	MID, HB, HF	35	23.7 ± 5.3	67.3 ± 11.2	167.7 ± 4.4	F	QWAFL (sub-elite)	2016	14	178
Boyd et al. [44]	MID, Nomadics, Deeps, Ruckman	19 21	25.2 ± 3.8 21.3 ± 2.4	87.9 ± 8.6 87.7 ± 18.4	1.87 ± 0.06 (m) 185.3 ± 17.2	M	AFL (elite) VFL (sub-elite)	2008/2009 2008/2009	24 29	NR NR
Clarke et al. [3]	MID, RUCK, SB, SF, TB, TF	23 36	23.8 ± 7.6 26.4 ± 4.5	68.2 ± 7.3 63.6 ± 6.8	171.2 ± 3.7 168.4 ± 6.9	F	AFLW (elite) VFLW (sub-elite)	2017	7 (av; 3.9) 13 (av; 7.3)	91 263
Clarke et al. [38]	MID, RUCK, SB, SF, TB, TF	26	23.8 ± 7.6	68.2 ± 7.3	171.2 ± 3.7	F	AFLW (elite)	NR	7	143
Corbett et al. [47]	NR	37	23 ± 4	86 ± 9	187 ± 8	M	AFL (elite)	2017	19	NR
Coutts et al. [4]	TB, MB, MID, TF, MF, RUCK	39	24.6 ± 2.9	88.7 ± 8.7	188.4 ± 7.2	M	AFL (elite)	2 seasons	19	342
Delaney et al. [48]	MID, MB, MF, RUCK, TB, TF	40	24 ± 3	87.9 ± 5.4	1.91 ± 0.04 (m)	M	AFL (elite)	2014/2015	30 (1–29 per player)	623
Edwards et al. [33]	NR	20	23.2 ± 2.7	83.3 ± 7	184.8 ± 5.5	M	WAFL (sub-elite)	2015	20	123
Garrett et al. [23]	NR	12 11	22.5 ± 4.2 22.3 ± 2.9	87.4 ± 6.8 80.9 ± 6.2	190.1 ± 6.5 184.4 ± 5.8	M	AFL (elite) SANFL (sub-elite)	NR	1	12 11
Gastin et al. [25]	NR	26	22.8 ± 3.3	85.8 ± 7.4	187.1 ± 7.2	M	AFL (elite)	1 season	8 (5 ± 2 per player)	126
Hiscock et al. [5]	Nomadics, Key Position	30	23.8 ± 3.3	88.3 ± 7.5	188.6 ± 7.5	M	AFL (elite)	2011	17	355
Johnston et al. [31]	MID, Fixed, Smalls	38	23.6 ± 4.5	89.7 ± 7.5	187 ± 17.1	M	AFL (elite)	2017	22	450
Johnston et al. [62]	Nomadic, fixed forward, fixed defenders, ruckman	30	24.2 ± 3.4	88.9 ± 9.1	187.3 ± 7.1	M	AFL (elite)	2011/2012	1–29 per player	419
Johnston et al. [36]	Nomadic	21	24.3 ± 3.7	84.9 ± 6.6	184.2 ± 5.8	M	AFL (elite)	2011/2012	1–22 per player	336
Johnston et al. [37]	Nomadic	23 26	24.3 ± 3.6 22.1 ± 2.9	84.8 ± 6.8 83.4 ± 5.9	184.4 ± 5.9 184.9 ± 6.7	M	AFL (elite) NEAFL (sub-elite)	2011/2012	30 31	336 164
Johnston et al. [20]	NR	21	24.4 ± 4	89 ± 8.9	1.86 ± 0.07 (m)	M	AFL (elite)	1 season	12 1–6 per player	69
Kelly et al. [35]	Nomadic and rotating positions	20 16	24.9 ± 3.8 21.7 ± 2.7	87.8 ± 9.4 84.9 ± 5.5	185.2 ± 6.9 184.6 ± 6.1	M	AFL (elite) Sub-elite	1 season	17 17	237 103
Kempton et al. [21]	Backs, MID, forwards	33	23.2 ± 1.8	87.3 ± 7.6	188 ± 7	M	AFL (elite)	2011/2012	31	511
Montgomery and Wisbey. [64]	Nomadic, forward, defenders	21	24.2 ± 1.7	86.3 ± 4.7	184.5 ± 3.5	M	AFL (elite)	Unsure	Unsure	Unsure
Mooney et al. [45]	NR	15	22.6 ± 3.2	84.3 ± 8.3	186.1 ± 6.5	M	AFL (elite)	1 season	22 1–4 per player	NR
Mooney et al. [29]	Key and non-key position	9	22.3 ± 3.3	86.5 ± 8.7	187.6 ± 7.3	M	AFL (elite)	1 season	5 1–3 per player	21
Rennie et al. [26]	NR	33	24.8 ± 4.2	88.3 ± 8.7	1.88 ± 0.8 (m)	M	AFL (elite)	1 season	18	360
Routledge et al. [27]	NR	42	24 ± 3	85 ± 8.1	188 ± 7.8	M	AFL (elite)	2017	22	NR
Stares et al. [30]	Nomadic and non-nomadic	30	24.3 ± 3.3	88.8 ± 8.4	189.1 ± 7.1	M	AFL (elite)	2014	16 ± 5 per player	NR
Stein et al. [34]	NR	14 10 16	24.9 ± 4.9 27.3 ± 5.2 27.4 ± 3.6	82.8 ± 7.7 82.6 ± 11.1 88 ± 8.42	182.1 ± 6.3 183.3 ± 7.2 182.8 ± 7.3	M	NEAFL (sub-elite) Recreational (grade) Recreational (reserve)	1 season	NR	26 23 32
Sullivan et al. [46]	NR	40	23.9 ± 3	87.7 ± 8.4	188.3 ± 7.2	M	AFL (elite)	2012	15 1–15 per player	292
Thornton et al. [28]	MID, Ruck, MB, MF, TF, TB	28	24.1 ± 4.9	68.3 ± 6.5	171.9 ± 6.7	F	AFLW (elite)	2020	7 (5.6 ± 2.1 per player)	140
Weston et al. [22]	NR	37	22 ± 3	84.4 ± 8.3	187 ± 7	M	AFL (elite)	2013	9 3–8 per player	129

Key: NR, not reported; MID, midfield; HL, half line; FL, full line; HB, half back; HF, half forward; SB, small back; SF, small forward; TB, tall back; TF, tall forward; AFL, Australian Football League; VFL, Victorian Football League; WAFL, West Australian Football League; SANFL, South Australian National Football League; NEAFL, North East Australian Football League; AFLW, Australian Football League Women’s; VFLW, Victorian Football League Women’s; QWAFL, Queensland Women’s Australian Football League

**Table 4:** Data Collection methods of included manuscripts

References	Microsensor device		Data collection				
	Brand	Model	Software	SF (Hz)	HDOP (*n*)	Satellites (*n*)	Metrics Reported
Aughey. [24]	Catapult	MinimaxX team sport 2.0	Logan Plus v4.1	5	NR	NR	TD, LIA, HIR, ACC
Aughey. [61]	Catapult	MinimaxX team sport 2.0	Logan Plus v4.1	5	NR	NR	TD, HIR, ACC
Bellinger et al. [32]	Catapult	MinimaxX team 2.5	Logan Plus v4.0	10	NR	NR	PL, TD, HSR
Black et al. [41]	Catapult	OptimEye S5	Sprint 5.1.7	10	NR	NR	TD, LS, MS, HS
Black et al. [39]	Catapult	OptimEye S5	Sprint 5.1.7	10	NR	NR	TD, LS, HS
Black et al. [40]	Catapult	OptimEye S5	Sprint 5.1.7	10	NR	NR	TD, LS, MS, HS
Boyd et al. [44]	Catapult	MinimaxX 2.0	Logan Plus v4.4	100*	NR	NR	PlayerLoad™
Clarke et al. [3]	Catapult	OptimEye S5	Openfield, 1.14.0	10	NR	NR	TD, LIA, HSR, VHSR, Sprint
Clarke et al. [38]	Catapult	OptimEye S5	Openfield, 1.14.0	10	0.9 ± 0.3	11.3 ± 0.8	TD, HSR, VHSR, Sprint, PlayerLoad™
Corbett et al. [47]	Catapult	T6 LPS and S5 GPS	Openfield v 1.11.2–1.13.1	10	0.6–1.5	> 8 packets per second	TD
Coutts et al. [4]	Catapult	Team Sport 2.5	Sprint v 5.0.6	10	NR	NR	TD, HSR, VHSR, Sprint, ACC, DEC
Delaney et al. [48]	Catapult	S5	Openfield v 1.12.0	10	NR	NR	TD, HSR, Av. Acc/dec
Edwards et al. [33]	GPSports	SPI Pro X	Team AMS	10	NR	NR	TD, HSR
Garrett et al. [23]	Catapult	MinimaxX Team 2.5	Sprint v 5.1.5	100*	< 2.0	≥ 8	TD, PlayerLoad™, HSR, VHSR
Gastin et al. [25]	Catapult	MinimaxX v4.0	Sprint 5	10	NR	NR	TD, Sprint distance, ACC, DEC, PL
Hiscock et al. [5]	GPSports	SPI Pro X	Team AMS-Release	15	NR	NR	TD, V1, Velocity load
Johnston et al. [31]	Catapult	OptimEye S5	Openfield v 1.15.0	10	NR	NR	TD, PlayerLoad™
Johnston et al. [62]	Catapult	Minimax X S3/ S4	Sprint 5.0.9	5 or 10	1 ± 0.3	12.2 ± 0.7	ACC, DEC
Johnston et al. [36]	Catapult	Minimax X S3/ S4	Sprint 5.0.9	5 or 10	1 ± 0.3	12.2 ± 0.7	TD, PlayerLoad™, LSR, HSR, ACC, DEC, HSR efforts
Johnston et al. [37]	Catapult	Minimax X S3/ S4	Sprint 5.0.9	5 or 10	1 ± 0.2	12.1 ± 0.7	TD, PlayerLoad™, LSR, HSR, ACC, DEC, HSR efforts
Johnston et al. [20]	Catapult	Team Sport 2.5	NR	5	NR	NR	TD, LSR, HSR, VHSR
Kelly et al. [35]	Catapult	NR	Sprint 5.1.6	10	NR	NR	TD, PlayerLoad™, LSR, HSR, ACC, DEC
Kempton et al. [21]	Catapult	Team Sport 2.5	Sprint v 5.0.6	10	NR	NR	TD, HSR, VHSR, Sprint, Sprint efforts, PlayerLoad™
Montgomery and Wisbey. [64]	Catapult	NR	NR	10	NR	NR	TD
Mooney et al. [45]	Catapult	MinimaxX Team 2.5	Logan Plus v 4.4.0	5	NR	NR	TD, HSR, LSA, ACC load
Mooney et al. [29]	Catapult	MinimaxX Team 2.5	Logan Plus v 4.4.0	5	NR	NR	TD, HIR
Rennie et al. [26]	Catapult	Optimeye S5	NR	10	1.1 ± 0.1	18.2 ± 1.1	TD, HSR, LSR, ACC, DEC
Routledge et al. [27]	Catapult	Optimeye S5	Openfield v 11.1.2	10	NR	NR	TD, HSR, Sprint, Running
Stares et al. [30]	GPSports	SPI-HPU	Team AMS-release 1	5**	NR	NR	TD, HIR, HSR, Sprint, ACC
Stein et al. [34]	Catapult	MinimaxX S4	NR	10	NR	NR	TD, LIA, MIA, HIA, ACC, repeat HIE
Sullivan et al. [46]	Catapult	Team Sport 2.5	Sprint v 5.0.6	10	1.25 ± 0.19	NR	TD, HSR, Sprint efforts, ACC, BodyLoad™
Thornton et al. [28]	Catapult	Optimeye S5	Openfield	10	0.64 ± 0.22	9.61 ± 1.70	TD, HSR, VHSR, ACC, ACC Load
Weston et al. [22]	Catapult	MinimaxX S4	NR	10	< 2.0	> 6	TD, HSR, LSR, PlayerLoad™

^*^Accelerometer only, **Interpolated to 15 Hz

Key: SF, Sampling frequency; HDOP, Horizontal dilution of precision; NR, Not reported; TD, Total distance; LIA, Low intensity activity; HIR, High intensity running; ACC, Accelerations; LS, Low speed; MS, Moderate speed; HS, High speed; GPS, Global positioning system; LPS, Local positioning system; HSR, High speed running; VHSR, Very high speed running; DEC, Decelerations; HIE, High intensity efforts; LSR, Low speed running

A large scope of playing position definitions were reported amongst the 33 included articles. These include specific groups (e.g., small backs) and broader playing groups, including; half line or small position players; tall, deep, fixed or key position players; and nomadic or rotating positions (midfielders, small forwards, and small defenders) [1]. Oftentimes, these broader classifications are utilised within research papers to overcome issues of small sample sizes [1]. For the purposes of this review, where no specific positions were reported it was assumed that data were pooled from all playing positions.

### Total Running Distances

**Fig. 2:**
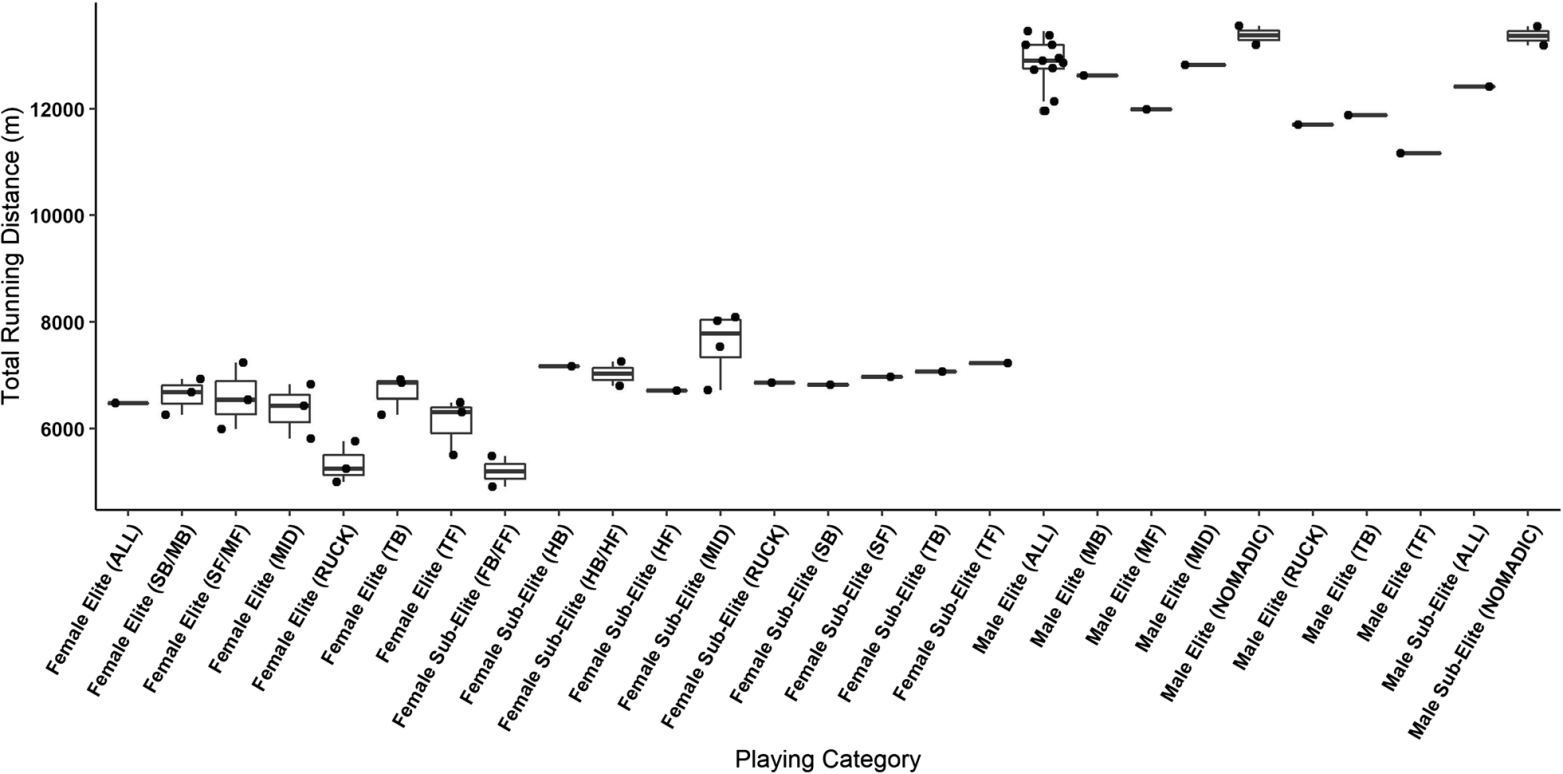
Comparison of running distances performed by male and female players. Boxplots represent distribution of the means of contributing manuscripts, lines represent mean when only one manuscript reported data for that sub-group, where black dots represent means from individual manuscripts. Key; MB: Mobile Back, MF: Mobile Forward, MID: Midfielder, SB: Small Back, SF: Small Forward, TB: Tall Back, TF: Tall Forward, FB/FF Full Back/ Full Forward combined, HB/HF: Half Back/Half Forward combined, HF: Half Forward, HB: Half Back

**Fig. 3:**
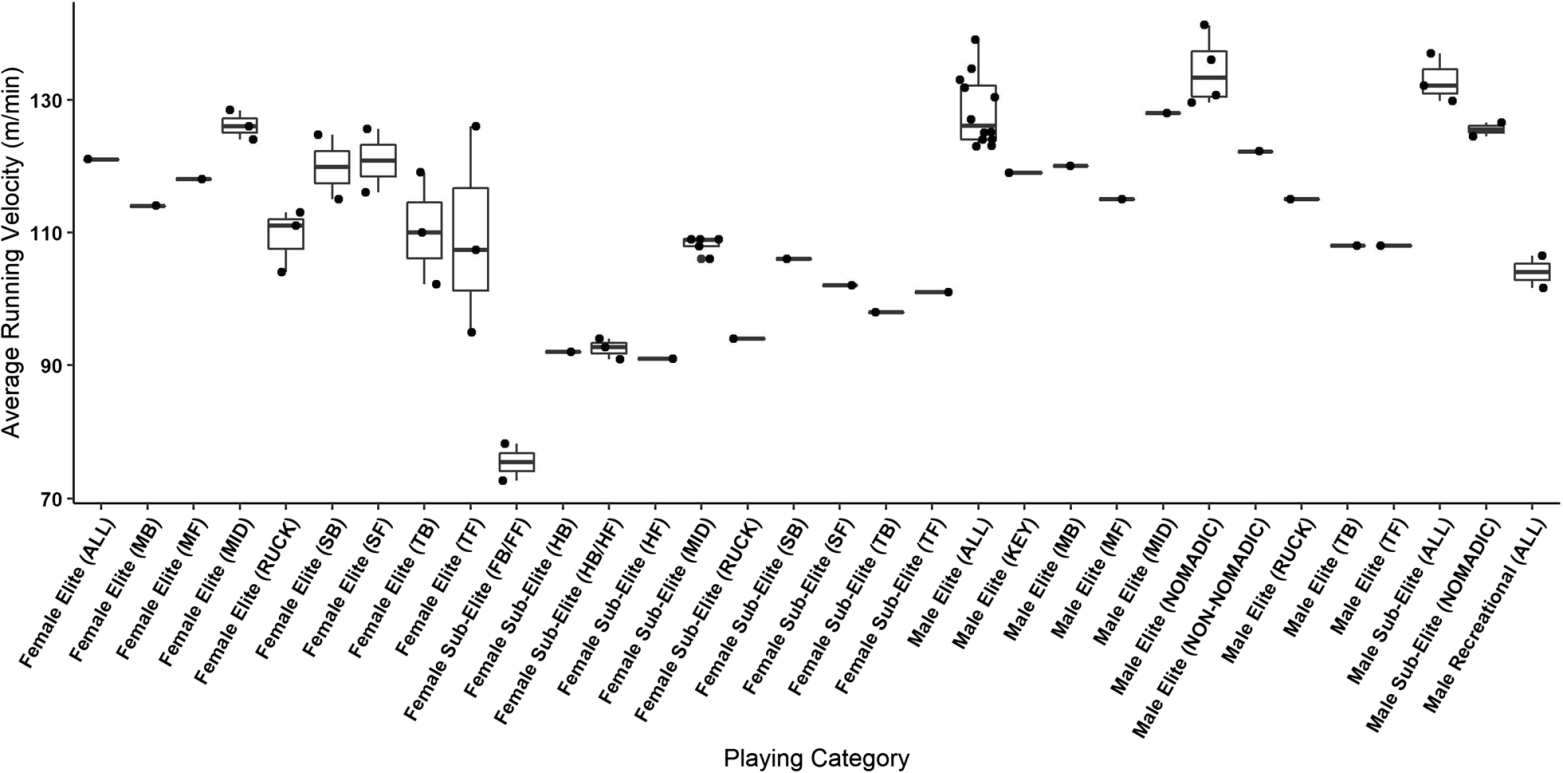
Comparison of relative running distances performed by male and female players. Boxplots represent distribution of the means of contributing manuscripts, lines represent mean when only one manuscript reported data for that sub-group, where black dots represent means from individual manuscripts. Key; MB: Mobile Back, MF: Mobile Forward, MID: Midfielder, SB: Small Back, SF: Small Forward, TB: Tall Back, TF: Tall Forward, FB/FF Full Back/ Full Forward combined, HB/HF: Half Back/Half Forward combined, HF: Half Forward, HB: Half Back, KEY: Key position

Total running distance was the most reported metric (see Figs. 2 and 3). When distances were pooled for all positional groups, male elite (range 13,455–11,954 m) [20–27] and sub-elite players (12,414.1 m) [23] recorded greater distances than female elite players (6474 ± 1013 m) [28]. These distances were also reported relative to playing time for male elite (range 139–123 ^−1^) [20–24, 26, 29–32], sub-elite (range 137–129.8 m·min^−1^) [23, 33, 34] and recreational players (range 106.5–101.6 m·min^−1^) [34], and for elite female players (121 ± 12 m·min^−1^) [28].

Running distances were also presented for male and female players delineated into positional groups. Amongst males, this included the nomadic and rotational positions at the elite (range 13,555.9–13,193.14 m, 141.2–129.6 m·min^−1^) [5, 30, 35–37] and sub-elite level (range 13,547–13,189.34 m, 126.53–124.5 m·min^−1^) [35, 37]. Coutts et al. [4] further divided these playing positions at the male elite level into midfielders (12,819 m, 128 m·min^−1^) mobile backs (12,621 m, 120 m·min^−1^), mobile forwards (11,986 m, 115 m·min^−1^), tall backs (11,878 m, 108 m·min^−1^), ruckman (11,701 m, 115 m·min^−1^) and tall forwards (11,158 m, 108 m·min^−1^). Additionally, Stares et al. [30] reported relative distances for male non-nomadic players (122.2 m·min^−1^), while Hiscock et al. [5] reported male key position players to reach 119 m·min^−1^.

Within female populations, data were presented for elite midfielders (range 6825–5813 m, 128.4–124 m·min^−1^), ruckman (range 5761–4998 m, 113–104 m·min^−1^), small or mobile backs (range 6926–6255 m, 124.7–114 m·min^−1^), small/ mobile forwards (range 7234–5996 m, 125.6–116 m·min^−1^), tall backs (range 6912–6255 m, 119–102.2 m·min^−1^), and tall forwards (range 6486–5506 m, 126–95 m·min^−1^) [3, 28, 38]. Data reported at the female sub-elite level included; midfielders (range 8087–6717 m, 109–106 m·min^−1^), half back (7167 m, 92 m·min^−1^), half forward (6706 m, 91 m·min^−1^), ruckman (6852 m, 94 m·min^−1^), small backs (6818 m, 106 m·min^−1^), small forwards (6964 m, 102 m·min^−1^), tall backs (7065 m, 98 m·min^−1^), tall forwards (7222 m, 101 m·min^−1^), half back and half forward groups combined (range 7249.7–6792.3 m, 94–90.9 m·min^−1^) and full back and full forward combined (5484.6–4909.8 m, 78.2–72.7 m·min^−1^) [3, 39–41].

### Running Distances Performed in Discrete Velocity Bands

**Fig. 4:**
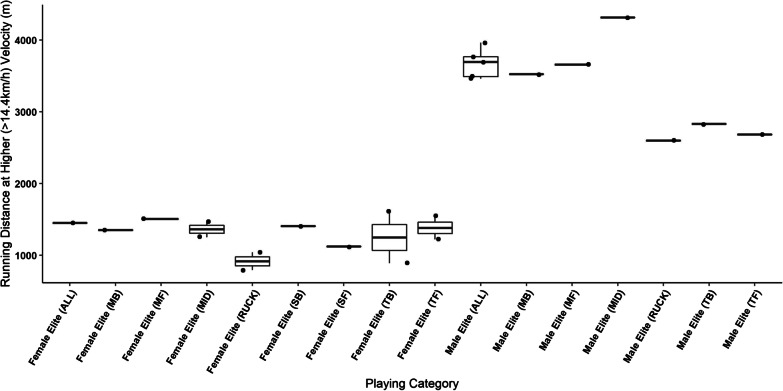
Comparison of high-speed running distances performed by male and female players. Boxplots represent distribution of the means of contributing manuscripts, lines represent mean when only one manuscript reported data for that sub-group, where black dots represent means from individual manuscripts. Key; MB: Mobile Back, MF: Mobile Forward, MID: Midfielder, SB: Small Back, SF: Small Forward, TB: Tall Back, TF: Tall Forward

Oftentimes, match running data are presented within discrete velocity bands (e.g., high speed running) which can enable practitioners to compare the proportion of an athlete’s total distance spent running at faster and slower speeds. However, the lack of a universally applied speed at which to categorise these velocity bands makes cross-study comparisons particularly challenging. Even so, a number of studies utilised 14.4 km/h (4 m.s^−1^) to define high-speed (or similar) speed zones for both male and female players, with males covering greater distances than females (see Fig. 4) [4, 21, 22, 26, 28, 38].

### PlayerLoad™

PlayerLoad™ is the summation of all accelerations recorded by the accelerometer component across all three movement axes (X = mediolateral, Y = anterior–posterior, Z = vertical), which represents the instantaneous change in acceleration, divided by a scaling factor and reported as an arbitrary unit (AU) [11, 42]. Therefore, PlayerLoad™ captures impacts from both foot strikes and body contacts, such as tackling and collisions [25, 43]. PlayerLoad™ was reported for male athletes at the elite (range 1413–1246 AU), and sub-elite level (1172.3 AU) [21–23, 25]. PlayerLoad™ was also reported relative to playing time at the male elite level for all positions (range 13.3–12.3 AU·min^−1^), midfielders (16.03 AU·min^−1^), ruckman (14.91 AU·min^−1^), deep position players (11.01 AU·min^−1^) and nomadic or rotating position players (14.96–12 AU·min^−1^) [23, 31, 32, 35, 36, 44]. Additionally, at the male sub-elite level, PlayerLoad™ relative to playing time has been reported for all positions (12.9 AU·min^−1^), as well as for midfielders (15.07 AU·min^−1^), ruckman (12.78 AU·min^−1^), deep position players (10.34 AU·min^−1^) and nomadic or rotating positions (13.03–12.1 AU·min^−1^) [23, 35, 37, 44].

Only one manuscript reported PlayerLoad™ for female athletes, and this was at the elite level. Clarke et al. [38] found small backs to record the highest absolute values (average; 629, 90% CI 533–710 AU), followed by midfielders (average; 599, 90% CI 533–663 AU), small forwards (average; 552, 90% CI 469–638 AU), tall backs (average; 477, 90% CI 405–550 AU), tall forwards (average; 477, 90% CI 398–556 AU) and ruckman (average; 448, 90% CI 336–559 AU). Utilising the playing durations provided by Clarke et al. [38], approximate PlayerLoad™ per minute was calculated as; midfielders (13.1 AU·min^−1^), small backs (12.5 AU·min^−1^), small forwards (11.3 AU·min^−1^), ruckman (9.9 AU·min^−1^), tall forwards (9.2 AU·min^−1^), and tall backs (8.9 AU·min^−1^).

### Match Periods

Several studies examined specific periods of a match. These included distances compared across playing quarters [3, 5, 45], with an assessment of winning versus losing quarters [5, 46]. Within male populations, the main decrement in running performance could be seen between quarters 1 and 4 [5, 45], whilst running demands were also greater in quarters lost [5, 46]. Elite female players also show the greatest reductions in running performance during quarter 4; however, running performance amongst sub-elite players tended to remain reasonably stable across the quarters [3].

Furthermore, peak periods of play, i.e.; time periods which identify the most intense running demands of the game, were also established within five of the included manuscripts [28, 31, 39, 47, 48]. Research within male AF demonstrated that peak periods were significantly greater than those reported using whole game data, and that the duration of the peak period had a significant impact upon running intensity, indicating that male AF players are exposed to short periods of high intensity running exercise [31, 48]. Similar findings have been demonstrated amongst female players, where peak period playing intensities were greatest over shorter analysis windows (e.g., 1-min), and those recorded when using whole game averaged data [28, 39].

## Discussion

### Total Running Distances

Data presented in this review highlights that, when playing positions are pooled, elite level male players cover approximately two times greater total running distance than their female counterparts [20–28]. This may, for the most part, be attributed to the differences in on-field playing time experienced by these athletes, with some female players competing for around 54 ± 10 min, whereas male athletes spend around 101 ± 12 min on ground [1, 28]. A similar trend was observed when assessing running distances with players delineated into the various playing positions, where male players covered greater distances than female players.

Interestingly, when distances are reported relative to playing time, differences are somewhat diminished. For example, Coutts et al. [4] reported male midfielders to cover more than double absolute running distances (12819 m, 95% CI 12,603–13034 m) than those highlighted within female midfielders (5813 m, 90% CI 5120–6505 m) in the report by Clarke et al. [38]. However, when expressed relative to playing time, there were no differences between the results (males; 128 m·min^−1^, 95% CI 126–130 m·min^−1^, females; 128.4 m·min^−1^, 90% CI 121.5–135.3 m·min^−1^) [4, 38]. The same results were also evident when making comparisons across the other playing positions highlighted within these two manuscripts [4, 38]. This finding not only demonstrates the potential comparative nature of male and female competitions, but also highlights the use of relative distances as a potentially more viable method when making comparisons across the two playing levels.

Additionally, it is valuable to compare those competing at different playing levels (e.g., elite vs sub-elite) as often those at the sub-elite level are drafted to the elite level competition, particularly within female AF. These comparisons can also inform physical performance pathways so that development players can be adequately prepared for elite level competition. Data presented within this review highlights that absolute total running distances performed within male AF matches is reflective of playing standard when playing positions are pooled together, with elite level players recording greater distances than sub-elite athletes [20–27]. However, when data for male elite and sub-elite athletes are delineated into playing positions, the differences between playing levels are not so clear. For example, Kelly et al. [35] found no significant differences between male elite and sub-elite nomadic and rotating position players (13,193.14 vs 13,189.34 m respectively). This was also evident when running distances were expressed relative to playing time where, in some cases, sub-elite level male athletes recorded higher meterage per minute than elite level athletes [23, 33, 34].

Amongst female players, there were contrasting results when comparing between playing levels [3, 38, 40, 41]. For example, of the six playing positions explored within the study by Clarke et al. [3], only female elite level midfielders and small forwards out-performed their sub-elite counterparts, potentially owing to the differences in playing time (elite 49 min, sub-elite 60 min). However, when these data were presented relative to playing time, there was a trend for an increase in running performance amongst the female elite level playing groups [3]. With these results in mind, it is possible that males performing at the sub-elite level are better prepared to perform at the intensity levels required at the elite level than females. Additionally, previous research has highlighted that the duration of sub-elite male AF matches is approximately 7 min longer than elite matches, potentially aiding development of match related running performance in sub-elite players [1]. However, it should be noted that Johnston et al. [1] reported elite level male players demonstrate superior performance in several measures of physical capacity to their sub-elite counterparts, inclusive of 3 km time trial, yo-yo intermittent recovery test, 20 m sprint and vertical jump, which should be considered when assessing the preparedness of sub-elite players to perform at the elite level. Additionally, it should also be noted that very few data exist at the male sub-elite level where players are delineated into discrete playing positions, which weakens our ability to make judgements of this nature.

Finally, it is common amongst male competitors for midfielders, nomadics, and small position players to cover greater distances (both relative and absolute) than tall and key position athletes [4, 5, 30, 35–37]. Johnston et al. [1] note that this is likely due to the requirement of midfielders and small position players to somewhat follow the ball, therefore utilising more of the playing oval, as opposed to tall and key position players whose role confines them to smaller sections of the ground. However, this trend was not always replicated within female populations, where there were some examples of tall and key position players out performing the midfield and small position players [3, 28, 38]. This finding may be attributed to sample size and player on-field time, which varies between the positions reported in the aforementioned studies [3, 28, 38]

These findings can enable practitioners to plan appropriate training volumes and intensities. Oftentimes, training load and intensity is prescribed based upon the physical requirements of the game and the position the player occupies. In this instance, the findings of this review suggest male players require higher running loads in order to adequately prepare for competition [20–28]. However, although female players seemingly require less overall volume of running based training (due to the reduced distances travelled in matches), the exposure to similar running intensities (i.e., relative distances) as their male counterparts appears desirable [4, 38]. This may be particularly relevant amongst sub-elite female players, where practitioners may wish to improve relative running performance/ running intensity in order to prepare female players for potential draft to the elite competition [3].

### Running Distances Performed in Discrete Velocity Bands

Due to the vast array of speeds used to define different velocity bands in the literature, cross-study comparisons were particularly challenging. However, what remains consistent across this body of research is that as velocity increases above high-speed or high-intensity running, the distance travelled decreases across all playing levels, and for both sexes, demonstrating the challenges faced by AF athletes in maintaining high-speed running outputs.

When studying high-intensity or high-speed running, distances covered at > 14.4 km·h^−1^ (> 4 m·s^−1^) were reported for elite male and female athletes [4, 21, 22, 28, 38], indicating that male athletes record greater distances above 14.4 km·h^−1^ (> 4 m·s^−1^) than female athletes across all positional groups, with elite male midfielders covering markedly greater distances (4314 m, 95% CI 4166–4462 m) [4] than elite female midfielders (1252 m, 90% CI 995–1508 m) [38]. These differences may be attributed to the increased ability of males to attain higher running velocities [28, 30], the differences in the style of play between the male and female game [49], and to the shorter game time in the female competition. However, when playing time is taken into consideration, Weston et al. [22] reported relative high-speed running distances to be 36 m amongst elite males, with the highest recorded for elite females seen amongst the midfield group as 28 m [38].

Additionally, an approximate 5–10% increase amongst male players was noted when calculating high-speed running as a percentage of total running volume. When all positions are pooled male athletes perform 26–33% of total running distances at a velocity > 14.4 km.h^−1^ (> 4 m.s^−1^), with females completing 22% at high speed [21, 22, 26, 28]. When athletes were delineated into their various playing positions, male midfielders and small or mobile position players performed around 8% more high-speed running relative to total distance than female midfielders and small/ mobile position players [4, 28, 38]. However, male and female tall and ruck position players performed much similar percentages at high-speed [4, 28, 38], further supporting the notion that positional role may play a significant role in the opportunity for these positional groups to perform high speed running [1].

As previously mentioned, the differences in the completion of high-speed running during AF matches may be explained by several factors. These include both the increased playing time experienced by male players and the more “open” style of play evident in the AFL, which lends itself to high-speed running, as opposed to the contested/ congested play evident within the AFLW [49]. Despite these limiting factors within the female game, the ability of male athletes to complete more high-speed running, given the same velocity threshold, is likely attributed to their ability to attain greater maximal running velocities during match play [28, 30]. Previous research in similar sports has demonstrated male athletes display superior physical qualities, inclusive of countermovement jump height, sprint speed and performance upon the yo-yo intermittent recovery test, potentially aiding their ability to repeatedly produce greater maximal velocity efforts [16]. Therefore, when the same speed is utilised to define high-speed running zones, it is likely that females will experience a higher physiological cost than their male counterparts [17].

As it has also been established that sprint performance is strongly associated with strength qualities, and therefore training status, the ability of female AF players to attain greater maximal velocities, and potentially increase their capacity to both complete and tolerate high-speed running distances, may be improved with greater exposure to training of this nature [50, 51]. This is particularly pertinent with elite female players who are reported to have a younger training age relative to their male counterparts, whilst also having reduced opportunity for training due to the part-time nature of the female game [28]. This is an important consideration, as greater pre-season training load (e.g., total and high-speed distances) has been associated with an increase in running performance during AF matches amongst male populations [52]. Furthermore, maximal aerobic running speed [53], 2-km time trial and yo-yo test performance [34], as well as measures of lower body power [30], have all been associated with running performance of male players. Therefore, in order to further enhance the female game, and to develop appropriate physical development pathways, it is a necessity that female athletes are afforded a greater opportunity to train.

Due to the reduced ability of female players to reach similar maximal velocities, a more accurate comparison may be made if high-speed running is defined utilising a percentage of maximal speed or similar physiological measurement. This method has been employed in female rugby sevens, where it was shown that a globally applied zone can under estimate high-speed running compared to one applied through the use of a physiological measure [54]. However, it should be recognised that applying a physiologically based threshold is not without its own complications, and requires further consideration [17]. It should also be noted that 14.4 km·h^−1^ (4 m·s^−1^) does appear to be reasonably slow to utilise as a measure of high-speed running, especially when it can be considered to be less than 50% of a male athlete's maximal velocity [30].

### PlayerLoad™

PlayerLoad™ was reported for male and female athletes across varying playing levels. Amongst male athletes, those at the elite level recorded higher values than their sub-elite counterparts [21–23]. The research by Clarke et al. [38] highlighted that female athletes recorded lower PlayerLoad™ volumes than male athletes, likely owing to the reduced playing time experienced by female players, and additionally, that midfielders and small position players perform a greater volume than tall position players. This was also noted within male populations, where Boyd et al. [44] reported midfielders and nomadics to record higher PlayerLoad™.min^−1^ than both ruckman and deep position players. PlayerLoad™ has been positively related to running distances, in part due to foot strike impacts contributing to the total load [25, 43]. Therefore, these findings are perhaps unsurprising, with male athletes and small position players having previously been shown within this review to cover greater running distances than female athletes and tall position players respectively. However, it is important to note that recent research has demonstrated PlayerLoad™ may underestimate actual player load by ~ 15%, highlighting the need for caution when utilising this metric in both research and practical settings [42]

### Match Periods

Previous research has demonstrated that using averaged data (e.g., total distance divided by total game time) can underestimate demands of intermittent type team sports [31, 39, 48, 55–57]. There has been a growing trend to identify the peak, or the most intense, periods of play within recent research [28, 31, 39, 47, 48, 55–57]. These periods have been established within AF, typically using a rolling-time frame approach [31, 39, 47, 48]. Peak periods of play could be seen to be as high as 1.8 times greater for meters per minute, and over 4 times greater for high-speed running per minute, than that recorded using whole game averaged data amongst female AF athletes [39]. Similarly, Johnston et al. [31] demonstrated within male populations that both meters and PlayerLoad™ per minute could rise to almost twice those seen using whole game averaged data during peak periods of play. In comparison, Thornton et al. [28] found that the peak 1 min period, recorded amongst elite female athletes, was reasonably similar to that recorded within male populations [31, 48]. However, the decline in physical output during 10 min periods was seen to be greater within female players, indicating that female athletes are not as able to maintain high intensity outputs over longer time periods [28]. Additionally, the peak period intensities highlighted by Thornton et al. [28] appear to be substantially higher than those found amongst sub-elite female athletes [39], highlighting a potential area for development amongst this population.

Delaney et al. [48] reported that, amongst male players, the highest demands during peak plays could be seen amongst the mobile forwards playing group. The review by Johnston et al. [1] speculated that, due to the playing position, these highly intense periods of play may be occurring during critical game moments (e.g., creating goal scoring opportunities). Although the contribution of high intensity actions to successful play has been somewhat established within soccer [58] and rugby union [59], to the knowledge of the authors this is yet to be established within AF populations, and therefore warrants further research. Furthermore, it was generally established within the included literature that the shorter the time frame analysed, the greater the demands were found, suggesting that stint duration has an effect upon the values recorded during peak periods for both sexes [31, 39, 47, 48]. It is important for both sports scientists and coaches to have an understanding of the demands of these shorter epochs and how to best prepare their athletes for these events [48, 60].

Match quarters [3, 5, 45, 46, 61] have also been investigated within AF populations. Decrements in running performance, for both males and females, were noted across quarters, with the greatest differences noted between quarter 1 and quarter 4, presumably indicating the increased impact of accumulated fatigue [3, 5, 45, 61]. Interestingly, Mooney et al. [45] demonstrated a very small, non-significant, increase in distance and high-speed running distance in quarter 3 in comparison to quarter 2 within a population of male players, possibly highlighting an effect of the half time break. It appears that within female AF populations, this decrement in running performance is accentuated at the higher velocity bands (e.g., sprint speed running), again highlighting the challenge facing AF athletes when attempting to maintain high-velocity outputs [3]. Finally, coaches can expect running outputs to be higher during quarters lost than quarters won [5, 46].

## Limitations

There are several limitations of this review that we acknowledge. Most pertinent is the difficulty in making cross-study comparisons due to the heterogeneity of metrics, such as different velocity bands and playing positions with a diversity of definitions used. Despite a large body of data for male players, there is comparatively little concerning female players. Similarly, there are also limited data with players separated into specific playing positions, with none reported for sub-elite male players. In some cases, only the results of one manuscript were reported for some sub-groups, which limits the strength of any comparisons made. Additionally, comparisons of accelerations and decelerations across the male and female players were not possible due to differences in methodologies across studies [28, 62]. Information of this nature would have been useful to further understanding of differences in running performance. Finally, there is an innate limitation when comparing male and female AF players, due to the contrasting match rules. This not only exists between male and female athletes but also between the elite and sub-elite levels of the female game. Nonetheless, comparisons of this nature are useful to practitioners in the field when devising training and load monitoring protocols across different playing groups. With these limitations in mind, future research should seek to develop a greater understanding of both female AF players and sub-elite male players. Particular emphasis should be placed upon both acceleration and decelerations as well as enhancing the depth of knowledge available when sub-elite male athletes are delineated into the various playing positions.

## Conclusion

This systematic review is the first to compare running performance between male and female AF players. The findings highlight male athletes record substantially higher running distances, and distances covered at high-speed, as well as PlayerLoad™ than female athletes during AF matches. This can be attributed to several factors including match duration, playing rules, and physical capacity. However, it is also likely affected by the greater opportunity afforded to male athletes to train. Despite male and female athletes being defined as “elite”, the female game is relatively young in nature whilst not yet a full-time occupation—as opposed to the elite level of the male game. This leads to greater training and performance opportunities for male athletes (e.g., the AFL season is typically 23 matches plus finals series, whilst the AFLW season is typically 7–9 matches plus finals series), which should be taken into consideration when making comparisons between these two groups of athletes [63].

When total running distances were expressed relative to playing time, it could be seen that the differences between male and female athletes were significantly reduced, indicating that female AF players can reach similar levels of running intensity. However, when peak periods of play were analysed, it was demonstrated that these could not be maintained to the same levels by female athletes once the analysis window was lengthened. Additionally, relative high-speed running, and high-speed running expressed as a percentage of total distance, remained comparatively reduced amongst female players. Practitioners in the field should be aware of these differences and similarities when planning both training volumes and intensities. In this respect, male players should be exposed to higher training volumes, whereas training intensities should be reasonably similar between male and female players.

## Practical Applications

1. To prepare for the current external loads of AF matches, female players may require lower training volumes, but similar relative intensities as male players.
2. Due to their enhanced ability to attain maximal running velocities, male athletes should have greater exposure to high-speed running (> 14.4 km·h^−1^ or > 4 m·s^−1^) during physical preparation periods. Additionally, there appears to be scope for improvement of high-speed running amongst female players should an increased opportunity to relevant training be afforded within AF programs and athletic development pathways.
3. Peak periods of play are similar between elite male and female AF players over shorter (e.g., 1 min) time periods, which may be reflected when prescribing drills aimed at replicating these phases of play, where similar running intensities appear to be appropriate.

## Data Availability

Not applicable.
